# Case Report: Proximal bronchial injury in small-cell lung cancer patient after moderately hypofractionated radiotherapy

**DOI:** 10.3389/fonc.2025.1566693

**Published:** 2025-05-19

**Authors:** Tongsong Zhang, Yun Wang, Haiqing Wang, Chao Yan, Chengcheng Dai, Linli Qu, Tao Yang, Aijie Yang

**Affiliations:** ^1^ Department of Radiotherapy, Qilu Hospital of Shandong University (Qingdao), Shandong University, Shandong, Qingdao, China; ^2^ Department of Radiotherapy, United Family Hospital Qingdao, Shandong, Qingdao, China; ^3^ Department of Oncology, Qingdao Central Hospital, University of Health and Rehabilitation Sciences (Qingdao Central Hospital), Shandong, Qingdao, China

**Keywords:** small-cell lung cancer, proximal bronchial tree, moderately hypofractionated radiotherapy, radiation-induced airway disease, atelectasis, bronchial stenosis

## Abstract

**Background:**

Proximal bronchial injury is a frequently observed complication in patients with central lung cancer following high-dose stereotactic body radiotherapy, whereas it is rarely reported after moderately hypofractionated radiotherapy. In this article, we present a case of proximal bronchial injury in a patient with small-cell lung cancer after moderately hypofractionated radiotherapy.

**Case presentation:**

A 45-year-old male patient with no history of smoking was diagnosed with limited stage small-cell lung cancer. According to guidelines of the National Comprehensive Cancer Network, the patient was treated with chemoradiotherapy, which included etoposide and cisplatin as well as radiotherapy at a dose of 65 Gy/26 fractions. Three months after radiotherapy, the tumor disappeared; however, stenosis of the right main bronchus, right upper lobe bronchus, and intermediate bronchus, as well as atelectasis of the right upper and middle lobes, occurred and gradually worsened. Anti-infection and hormonal therapy were ineffective. One year after radiotherapy, grade 3 damage was formed in the proximal bronchus according to the Common Terminology Criteria for Adverse Events (version 5.0). Following endoscopic balloon dilatation of the right main bronchus, asthma symptoms of the patient were reduced.

**Conclusion:**

This case reminds us that it is necessary to implement a proximal bronchial dose constraint and prevent the occurrence of dose hot spot in the proximal bronchus when administering moderately hypofractionated radiotherapy with a physical dose exceeding 65 Gy.

## Introduction

Lung cancer is the second most common type of malignant tumors and the leading cause of mortality in humans (1). Small-cell lung cancer (SCLC) accounts for 10%–15% of all lung cancer cases (2). It is a neuroendocrine tumor characterized by easy metastasis, a poor prognosis, and a high mutation load (3). Chemoradiotherapy combining the administration of etoposide and cisplatin for 4–6 cycles with chest radiotherapy (RT) and preventative brain irradiation remains the standard modality for the treatment of limited stage SCLC (LS-SCLC) (4, 5). SCLC tumor cells have a short doubling time. Thus, hyperfractionated radiotherapy (Hyper-RT) and hypofractionated radiotherapy (Hypo-RT) have been utilized to reduce the repopulation of these rapidly proliferating tumor cells. Because Hyper-RT causes serious esophagus acute radiation injury, an increasing number of studies have focused on Hypo-RT for the treatment of SCLC.

The tracheobronchial structures exhibit reduced radiosensitivity compared to the alveolar epithelium within the pulmonary parenchyma. In most patients, the standard dose of RT (60–66 Gy) typically does not cause damage to the airway. Although high-dose RT improves local tumor control, it is associated with life-threatening side effects (6). Radiation-induced airway damage, also termed radiation-induced airway disease (RIAD), is a long-term toxicity problem. It includes central airway stenosis, atelectasis, necrosis, and severe radiation-induced toxicity, and may even lead to death. The airway refers to the proximal bronchial tree (PBT), which includes the distal 2 cm of the trachea, bulge, bilateral main bronchus, bilateral upper lobe bronchus, middle bronchus, right middle lobe bronchus, lingual segment bronchus, and bilateral lower lobe bronchus. PBT injury is frequently reported after stereotactic body RT, whereas it is rarely reported following moderately Hypo-RT (7, 8). In this article, we report a case of PBT injury after moderately Hypo-RT.

## Case presentation

**Figure 1:**
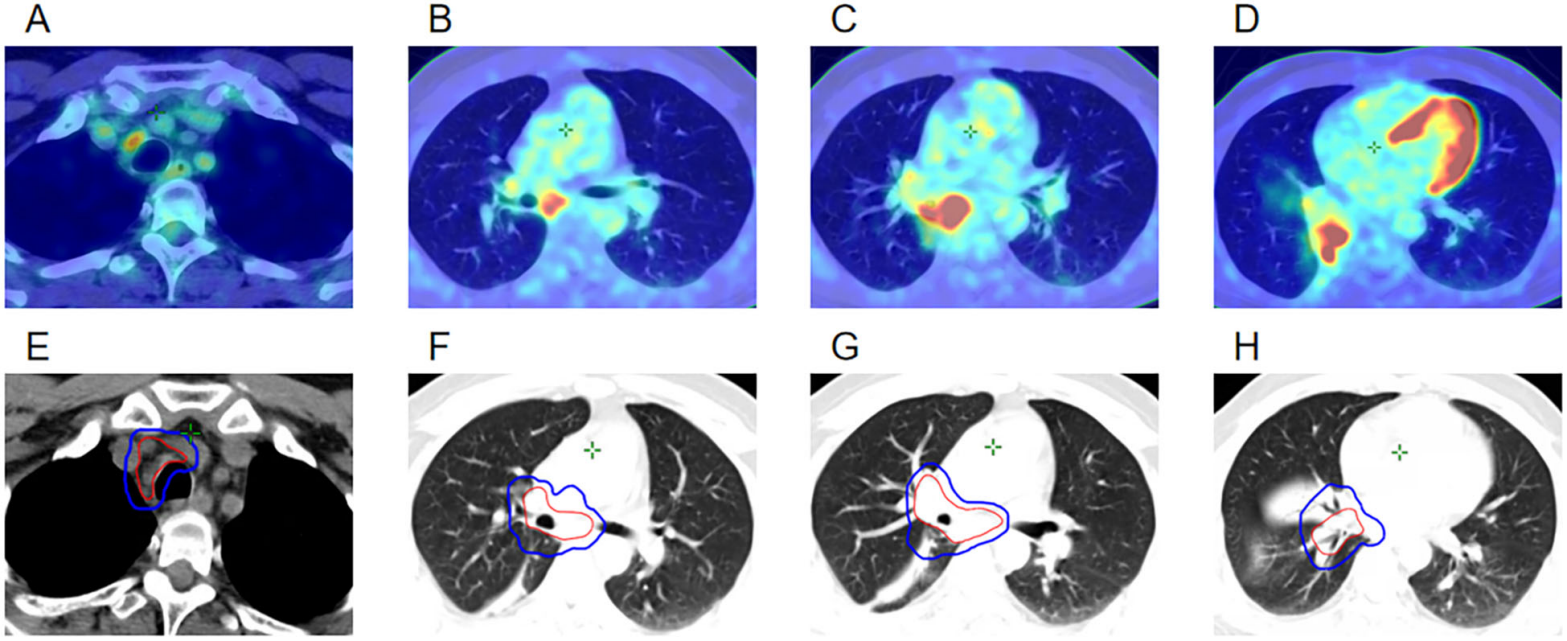
**(A–D)** PET-CT images at the time of initial diagnosis in March 2023. **(E–H)** Clinical target volume and planning target volume of the radiotherapy target area in April 2023.

**Figure 2:**
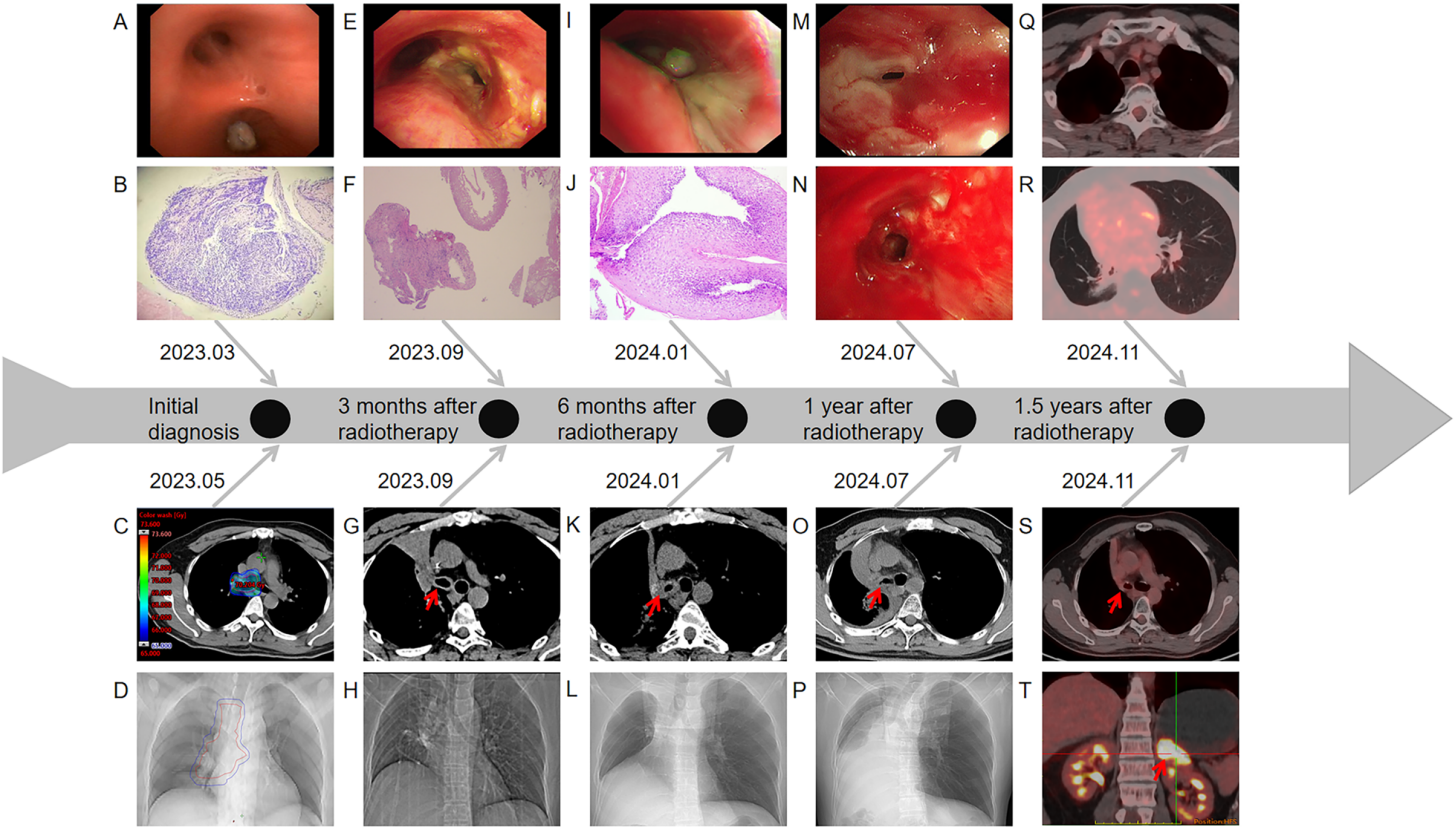
**(A)** Image of the tumor detected on the first bronchoscopy (March 2023). **(B)** Pathological results of the tumor in the right intermediate bronchus (March 2023). **(C)** Dose distribution in the target area at the carina. **(D)** Chest CT localization images before radiotherapy. **(E)** Image of the carina on the second bronchoscopy (September 2023). **(F)** Pathological results of the right middle lobe bronchus on the second bronchoscopy (September 2023). **(G)** Chest CT images showing changes of the carina (September 2023). **(H)** Chest CT localization images (September 2023). **(I)** Image of the carina on the third bronchoscopy (January 2024). **(J)** Pathological results of the right main bronchus on the third bronchoscopy (January 2024). **(K)** Chest CT images showed changes of the carina (January 2024). **(L)** Chest CT localization images (January 2024). **(M)** Image of the opening of the right main bronchus on the fourth bronchoscopy (July 2024). **(N)** Image of the opening of the right main bronchus after balloon dilatation (July 2024). **(O)** Chest CT images showing changes of the carina (July 2024). **(P)** Chest CT localization images (July 2024). **(Q)** Image of the mediastinal window on PET-CT (November 2024). **(R)** Image of the lung window on PET-CT (November 2024). **(S)** PET-CT imaging showing changes of the carina after balloon expansion surgery (November 2024). **(T)** PET-CT indicated left adrenal metastasis (November 2024).

The patient was a Chinese non-smoker male patient (age: 45 years; Eastern Cooperative Oncology Group score: 0) with no medical history and no family history of cancer. He was admitted to the hospital due to low-grade fever, chest tightness, and cough in March 2023. Positron emission tomography-computed tomography (PET-CT) showed a hypermetabolic mass in the right lower lobe, hypermetabolism in the mediastinum and right hilar lymph nodes, and undistension of the right lower lobe (**Figures 1A–D**). Bronchoscopy showed a mass in the intermediate bronchus (**Figure 2A**), and biopsy confirmed small-cell carcinoma (**Figure 2B**). From March 2023 to July 2023, five cycles of chemotherapy with etoposide and cisplatin (etoposide: 0.1 g [days 1–5] + cisplatin 40 mg [days 1–3], 21 days/cycle) were administered according to guidelines of the National Comprehensive Cancer Network (NCCN). During the 2–3 cycles of chemotherapy, moderately Hypo-RT was administered. The RT protocol was as follows. Target volume delineation was performed using respiratory-correlated four-dimensional CT. The gross tumor volume encompassed all ^18^F-fluorodeoxyglucose-avid primary lesions and metastatic lymph nodes (short-axis ≥1 cm on diagnostic CT. The clinical target volume included gross tumor volume with 5 mm isotropic expansion while respecting anatomical barriers, plus elective nodal stations according to the consensus of the European Society for Radiotherapy and Oncology Advisory Committee on Radiation Oncology Practice (9). An internal target volume was generated through deformable registration of clinical target volume contours across 10 respiratory phases (0%–90% phase bins), validated against maximum intensity projection datasets. The planning target volume was defined as internal target volume plus 5 mm isotropic margin. Organ at risk dose constraints were: spinal cord: maximum dose (Dmax)<41 Gy; lungs (bilateral): V20 (percentage volume receiving > 20 Gy) ≤25%; mean lung dose ≤15 Gy; heart: V40 (percentage volume receiving > 40 Gy) ≤20%, mean heart dose ≤20 Gy; esophagus: mean dose ≤34 Gy, Dmax ≤66 Gy, and V60 (percentage volume receiving > 60 Gy) ≤17%. A volumetric modulated arc therapy plan was optimized to deliver: prescription dose: 65 Gy/25 fractions to ≥95% of the planning target volume; biological effective dose: 81.2 Gy (calculated per the linear-quadratic model, α/β = 10 Gy). Clinical target volume and planning target volume in RT target area are shown in **Figures 1E–H**, **2C–D**. The patient completed the planned chemotherapy and RT as scheduled, with good tolerance, no significant adverse effects, and a satisfactory clinical response. One month after RT (June 2023), the patient experienced mild cough, phlegm production, and wheezing, which progressively worsened. The symptoms improved after treatment with anti-infection agents and prednisone (40 mg/day). In September 2023, chest CT revealed a bronchial occlusion in the upper lobe of the right lung (**Figure 2H**) and thickening of the right main bronchial wall (**Figure 2G**). Few days later, bronchoscopy showed (**Figure 2E**) a number of white cheese exudates at the beginning of the right main bronchus and almost completely blocked upper and middle lobes of the right lung. Pathological biopsy of the right middle lobe showed (**Figure 2F**) absence of an epithelial structure in the tissue and presence of cellulose and inflammatory exudate. Prednisone dose was reduced to one tablet (5mg) every 4 days, then his discomfort symptoms were relieved. The lesions in the lower lobe of the right lung were effectively controlled; hence, the efficacy was evaluated as complete response. In November 2023, the patient received brain prophylactic irradiation protecting the hippocampus (RT total dose: 25 Gy/10 fractions). In January 2024, the patient developed a cough and phlegm production. Chest CT revealed thickening of the right main bronchial wall (**Figure 2K**) and obstructive atelectasis in the middle lobe (**Figure 2L**). Bronchoscopy was performed on January 2024, revealing that the trachea and carina were congested, with the surface covered in yellow and white moss, lumen congestion, and edema. Additionally, the right lung main bronchus was narrowed (**Figure 2I**). Pathology of the right main bronchus showed (**Figure 2J**) a few squamous epithelial mucosa, scattered with chronic inflammatory cells, focal submucosal infiltration of lymphocytes and plasma cells, and some necrotic tissue with inflammatory exudation. After 2 weeks of anti-infection and hormonal therapy, the symptoms improved slightly. Subsequently, the patient underwent regular follow-up examinations. On July 2024, the patient experienced chest tightness and shortness of breath. Chest CT showed narrowing of the main bronchus (**Figure 2O**), bronchial occlusion in the upper, middle, and lower lobes of the right lung, and aggravated obstructive atelectasis (**Figure 2P**). On July 2024, bronchoscopy was performed, which showed almost complete occlusion of the right main bronchus, scar formation, and granulation (**Figure 2M**). He underwent bronchoballoon dilatation, resulting in significant improvement of symptoms (**Figure 2N**). In November 2024, PET-CT showed that the narrowed right main bronchus had improved (**Figure 2S**), without findings of the primary lesion and mediastinal metastatic lymph nodes (**Figures 2Q, R**). However, he had developed left adrenal metastasis and underwent systemic chemotherapy with etoposide and cisplatin (**Figure 2T**).

**Figure 3:**
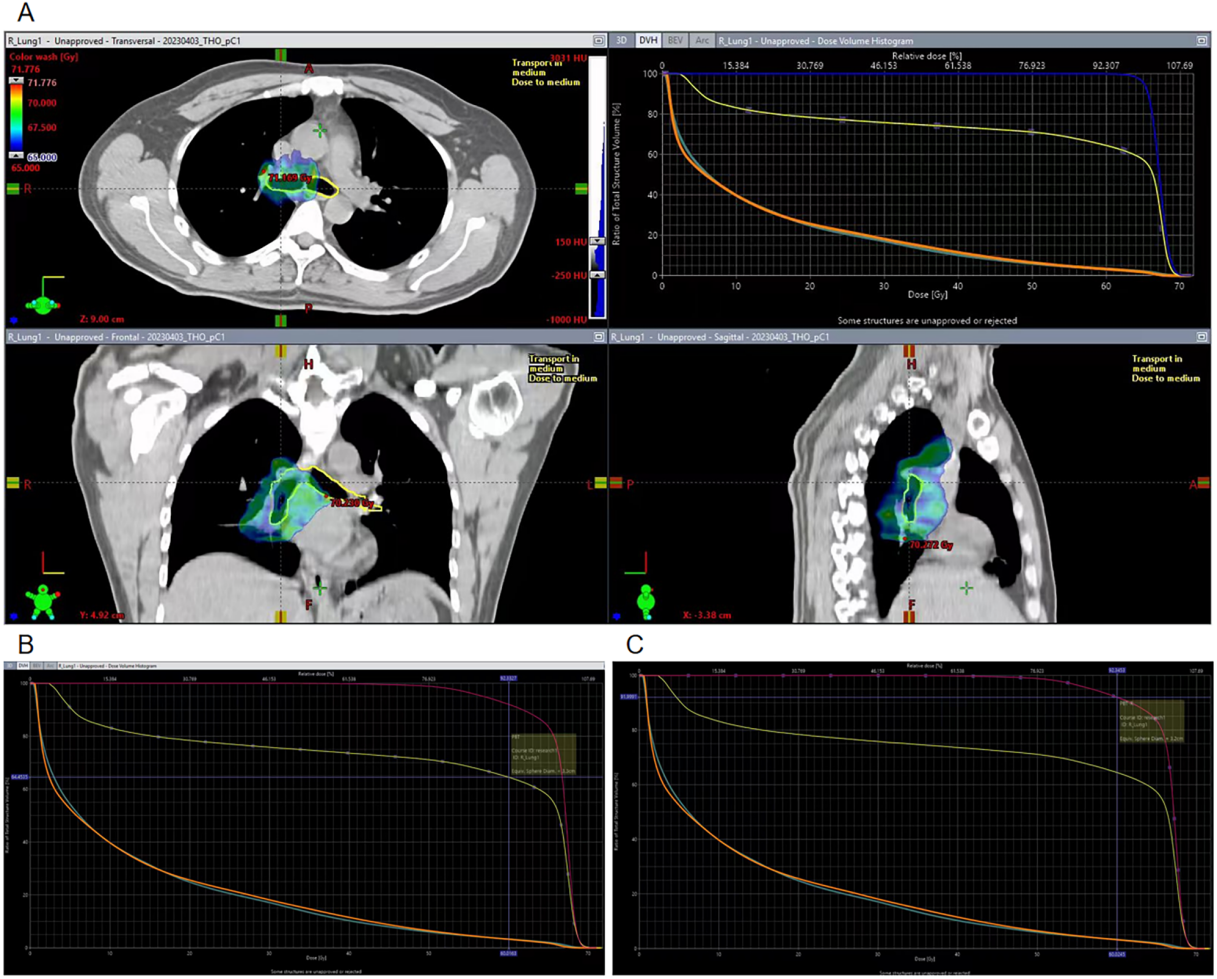
Target region dose images on the PBT and DVH chart. **(A)** Target region dose of the PBT and DVH chart. **(B)** V60 of the whole PBT in the DVH chart. **(C)** V60 of the right PBT in the DVH chart.

In this case, we did not delineate the PBT previously, because the total dose did not exceed 66 Gy. After the PBT lesion occurred, we redelineated the entire PBT and the right PBT, based on the original RT localization image and plan (**Figure 3A**). The dose-volume histogram chart revealed the following: entire PBT (**Figure 3B**): Dmax: 71.1 Gy, V60 (percentage of PBT volume receiving > 60 Gy): 64.5%, The right PBT (**Figure 3C**): Dmax: 71.1 Gy, V60: 91.9%.

## Discussion

The RT regimen recommended by the NCCN guideline for the treatment of LS-SCLC is Hyper-RT with split dose of 1.5 Gy twice-daily (bid) (total dose: 45 Gy) or conventional RT with split dose of 1.8–2 Gy once daily (total dose: 60–66 Gy) (4). SCLC demonstrates rapid tumor cell proliferation kinetics, characterized by accelerated doubling times and elevated mitotic indices. These biological features render it particularly susceptible to Hyper-RT and Hypo-RT. In a phase III clinical trial, the investigators increased the RT dose to 54 Gy/30 fractions bid; high-dose RT improved overall survival in patients with LS-SCLC compared with standard dose thoracic RT (45 Gy/30 fractions bid) (10). A phase II trial showed that higher doses of hyperfractionated, accelerated, twice-daily RT with 60 Gy/30 fractions bid significantly improved 2-year and median overall survival compared with the standard 45 Gy/30 fractions bid regimen (11). However, Hyper-RT is associated with a significantly higher risk of grade 3 acute radioactive esophagitis than conventional RT (32% vs. 16%, respectively), affecting patient compliance (12, 13). Additionally, Hyper-RT is inconvenient for RT institutions; thus, it has not been widely used in China. Hypo-RT increases the efficacy of a single dose, reduces the overall number of fractions required, and shortens the treatment cycle, aligning with the rapid tumor proliferation kinetics of SCLC, particularly its characteristically abbreviated cellular doubling time. Phase II randomized trials support the therapeutic potential of moderate hypofractionation in LS-SCLC. Qiu et al. compared once daily concurrent chemoradiotherapy (65 Gy/26 fractions) with conventional twice-daily concurrent chemoradiotherapy (45 Gy/30 fractions) in patients with LS-SCLC (Eastern Cooperative Oncology Group score: 0–1) (14). The hypofractionated regimen achieved significantly improved 2-year progression-free survival (42.3% vs. 28.4%, respectively, p = 0.031), while maintaining comparable rates of grade ≥3 toxicities: radiation pneumonitis (2.4% vs. 3.3%, respectively) and esophagitis (15.3% vs. 17.4%, respectively). Other toxicities, including pulmonary toxicity, were comparable between the two groups, with no proximal bronchial toxicity reported in either cohort. Additionally, no dose-limiting requirements for proximal bronchial toxicity were identified in either group. The linear-quadratic model showed that this regimen (65 Gy/26 fractions) achieved a higher biological tumor dosage (biological effective dose: 81.2 Gy), comparable to 66 Gy/33 fractions (biological effective dose: 79.2 Gy), while maintaining similar late toxicity profiles (α/β = 3 Gy for normal lung tissue). Following multidisciplinary review, three evidence-based treatment strategies were presented to the patient, including efficacy profiles, anticipated toxicities (acute/late), and long-term survivorship. The patient elected the Hypo-RT protocol. However, Hypo-RT use is not validated by large phase III randomized controlled clinical trials. Tjong et al. reviewed the current status and progress of RT management for SCLC, noting that controversy remains regarding the total dose and fractionation patterns for LS-SCLC; hence further investigation is required (15).

The therapeutic efficacy of RT is proportional to the dose. RIAD is rare in most patients at standard RT dose (60–66 Gy); however, with the increase of the total dose, the risk of RIAD also rises (16, 17). The pathophysiological mechanism of radiation-induced airway damage remains unclear. Radiation causes endothelial injury, aseptic inflammation, fibrosis, and necrosis in normal tissues, and leads to DNA damage, apoptosis, and microvascular damage (18). Radiation-induced bronchial injury may result in stenosis, fibrosis, and subsequent atelectasis, characterized by regional lung collapse and heightened lung density (19). This is consistent with the pathology observed on bronchoscopy in this case after injury. Three months following RT, sterile inflammatory exudation occurred, followed by fibrosis. One year after RT, scars and granulation tissue formed. According to the National Cancer Institute Adverse Event Evaluation Criteria version 5.0 (Common Terminology Criteria for Adverse Events version 5.0 [CTCAE v5.0]), RIAD is categorized into five distinct grades. The progression of RIAD involves an initial redness of the airway mucosa and thickening of bronchial secretions, which can lead to bronchial stenosis, cough, wheezing, recurrent infections, bronchial necrosis, bronchial fistula, and bronchopulmonary vascular hemorrhage resulting in hemoptysis. These severe manifestations can culminate in life-threatening respiratory and hemodynamic complications, necessitating urgent interventions, such as intubation or emergency treatment. In this case, the thickening of bronchial secretions and bronchial stenosis, which necessitated endoscopic treatment, denoted grade 3 damage, according to the CTCAE v.5.0. In the management of radiation-induced airway injury, it is imperative to account for concomitant treatment-related toxicities, including chemotherapy and immunotherapy, occurring during or following RT. The ADRIATIC study showed for the first time that consolidation therapy with durvalumab, administered after the completion of concurrent chemoradiotherapy for LS-SCLC, significantly enhanced progression-free and overall survival (20). However, consolidation immunotherapy was not administered to this patient due to grade 3 airway toxicity after RT. Moreover, subsequent systemic treatment for adrenal metastasis did not include immunotherapy. After four cycles of chemotherapy, local RT was planned for the adrenal metastatic lesion.

Based on the 2024 NCCN Guidelines, dose constraints for normal organs in SCLC are as follows. Under conventional fractionation (1.8–2.0 Gy per fraction), the dose constraints for critical organs should be adjusted according to tumor size and location, following principles similar to those for non-small cell lung cancer (NSCLC): spinal cord: Dmax<50 Gy; lungs (bilateral): V20 ≤35%, mean lung dose ≤20 Gy; heart: V40 ≤20%, mean heart dose ≤20 Gy; esophagus: mean dose ≤34 Gy, Dmax ≤105% of prescription dose and V60 ≤17%. For Hyper-RT or regimens with lower total doses, stricter constraints should be applied. There is currently no universally established consensus on standardized dose constraints for normal organs in Hypo-RT due to limited clinical data. Current recommendations are primarily extrapolated from conventional fractionation studies or institutional protocols, with ongoing research aiming to refine evidence-based guidelines for hypofractionated schedules. When employing Hypo-RT, the spinal cord Dmax should adhere to ≤41 Gy as defined in the CALGB 30610/RTOG 0538 trial, while conventional fractionation constraints should be followed for other organs, with priority given to minimizing dose exposure (21–23). Dose-volume histogram parameters for normal organs in this case were: spinal cord: Dmax = 39.4 Gy; lungs (bilateral): V20 = 24.8%, mean lung dose = 13.9 Gy; heart: V40 = 11.8%, mean heart dose = 14.3 Gy; esophagus: mean dose = 23.2 Gy, Dmax = 68 Gy, V60 = 10.9%. NCCN guidelines only specify the PBT as a dose-constrained organ at risk in stereotactic body RT planning. To prevent radiation-induced damage to the PBT, it is crucial to set a dose constraint; this constraint is established through practical experience. Miller et al. conducted high-dose Hyper-RT on patients with lung cancer who did not receive concurrent chemotherapy. They found that symptomatic airway stenosis was rare when the prescribed dose was< 70 Gy. However, when the prescribed dose increased to 74 Gy and 86 Gy, the incidence of airway stenosis rose to 4% and 25%, respectively. This stenosis occurred from 2 months to 4 years after RT, with the incidence of airway stenosis at 1 year and 4 years being 7% and 38%, respectively (16). Kelsey found that, in patients undergoing high-dose hyperfractionated external beam RT (prescribed dose ≥73.6 Gy), airway stenosis first appeared 3 months after RT. The caliber of the two irradiated main bronchi significantly decreased, showing a dose-dependent pattern, and this was more pronounced in patients receiving concurrent chemotherapy (24). The timing of the occurrence of bronchial stenosis in this case is consistent with this finding. Lee et al. prospectively analyzed 88 patients with NSCLC who received a prescribed dose ≥66 Gy (25). Among them, 21 patients (24%) developed late complications including late airway-related injuries, which occurred 2–13 months after RT. Notably, three (3.4%) and two (2.3%) patients developed bronchial stenosis and fatal hemoptysis, respectively. Those who developed fatal hemoptysis received prescribed doses of 82 Gy/41 fractions and 90 Gy/45 fractions, respectively. In this patient with LS-SCLC, a Hypo-RT of 65 Gy/26 fractions was selected to achieve better therapeutic effects, based on the phase II study (14). Ultimately, a progression-free survival of 1.5 years was attained. After synchronous chemoradiotherapy, the lesions in the lower lobe of the right lung were effectively controlled, and the efficacy was evaluated as complete response. However, the lung injury and the damage of the proximal airway after RT attracted our attention. The patient experienced recurrent lung infections and lung atelectasis in the right middle lobe 1 month after completing RT. During 1 year following treatment, the damage associated with RT has progressively worsened, culminating in grade 3 bronchial stenosis toxicity based on the CTCAE v5.0.

The majority of SCLC cases are categorized as centrally located lung tumors, arising from the proximal bronchial epithelium. Consequently, the target volume for therapeutic intervention must encompass the neoplastic bronchial lesions. While the anatomical constraints and dose-limiting toxicity thresholds of PBT have been systematically addressed in contemporary stereotactic body RT consensus guidelines, dose constraints under Hypo-RT regimens remain inadequately characterized in current thoracic oncology literature (26). Wang et al. reported that, in patients undergoing conventional RT, the median time from the onset of RT was 8.4 months, and the high-dose area of the standard equivalent dose (EQD_2_) was correlated with radiation-induced central airway toxicity (27). According to the formula: $EQD_{2}=nd\frac{d\;+\;\alpha \;/\;\beta }{2\;+\;\alpha \;/\;\beta }$ , none of the patients receiving EQD_2_ <65Gy experienced PBT toxicity; PBT V75<11.9% could be used to limit grade >2 PBT toxicity. They documented that 88 NSCLC patients (88%) received concurrent chemotherapy, but did not specify the chemotherapy regimens utilized. According to NCCN guidelines, the concurrent chemoradiation regimens for NSCLC include pemetrexed (adenocarcinoma) or taxane-based or etoposide agents combined with platinum-based agents. Notably, etoposide-based regimens are associated with lower pulmonary toxicity compared to taxane-based therapies. In this case, when using a prescription dose of 65 Gy/26 fractions (EQD_2_: 67.7 Gy). The hot spot dose is defined as 108% –110% of the prescribed dose. The hot spot dose was 71.1 Gy (EQD_2_:75.4 Gy). Despite adherence to conventional dosimetric constraints (V75<11.9%), the persistence of grade 3 PBT toxicity (CTCAE v5.0) was observed in this case where the EQD_2_ at the maximum point dose (Dmax) exceeded 75 Gy. It may be necessary to establish stricter dose constraints for PBT. There is limited research on the dose constraints of PBT in the hypofractionated mode. When implementing an unconventional fractionation of >65 Gy, it is necessary to delineate the PBT and impose dose constraint. During planning, caution should be exercised to avoid dose hot spot on bronchial openings. If the target area is unilaterally situated, it is advisable to precisely outline and restrict the affected-side bronchial tree.

When radiation leads to bronchitis and fibrosis, followed by bronchial stenosis or even occlusion (28), surgical treatment of bronchial stenosis is generally not used due to high rates of surgical complications and mortality (29). Non-surgical treatment measures (e.g., stent placement and balloon dilatation) can be attempted. In this case, the patient underwent bronchial balloon dilatation, which led to relief from wheezing. A follow-up bronchoscopy demonstrated a decrease in the degree of stenosis in the right main bronchus. Nevertheless, executing surgical procedures within a compromised bronchus entails the risk of hemorrhage; hence, rigorous case selection is necessary.

This is the first reported case of irreversible bronchial injury resulting from moderate-dose fractionated RT (65 Gy/26 fractions) leading to bronchial stenosis, occlusion, and atelectasis of the lung lobe, with an earlier onset and progressive worsening over time. RIAD is often overlooked in chest RT. With advancements in RT technology, the prescribed dose is frequently increased to achieve better tumor control, and the mode of dose fractionation in RT is becoming more varied. However, comprehensive evaluation of the radiation dose for the PBT remains a challenge. Conversion to EQD_2_ values can be used to more effectively compare the exposure dose and tolerance dose for the PBT across various segmentation modes. Currently, there is no clear data on the dose constraints for the PBT in unconventional fractionated RT. According to this case, when the total dose exceeds 65 Gy, we should delineate and constrain the entire and affected-side bronchial tree, while ensuring that dose hot spot do not occur in the PBT.

Based on the Robert Timmerman dose-volume constraints (version 2021.8) for 30-fraction regimens, we propose standardizing the prescription dose of Hypo-RT using EQD_2_ normalization, with dose-volume parameters constrainted to Dmax< 69 Gy and V60< 5 cc (30). The feasibility of modifying these constraints requires prospective validation through rigorously designed clinical trials.

In conclusion, for central lung cancer, it is crucial to safeguard the PBT and enforce restrictions to minimize the risk of RIAD, particularly when employing high-dose segmented RT.

## Data Availability

The original contributions presented in the study are included in the article/supplementary material. Further inquiries can be directed to the corresponding author.
